# Anti-predator meshing may provide greater protection for sea turtle nests than predator removal

**DOI:** 10.1371/journal.pone.0171831

**Published:** 2017-02-10

**Authors:** Julie M. O’Connor, Colin J. Limpus, Kate M. Hofmeister, Benjamin L. Allen, Scott E. Burnett

**Affiliations:** 1 University of the Sunshine Coast, Sippy Downs Drive, Sippy Downs, Queensland, Australia; 2 Sunshine Coast Regional Council, Caloundra, Queensland, Australia; 3 Department of Environment & Heritage Protection, Threatened Species Unit, Brisbane, Queensland, Australia; 4 University of Southern Queensland, Institute for Agriculture and the Environment, Toowoomba, Queensland, Australia; James Cook University, AUSTRALIA

## Abstract

The problem of how to protect sea turtle nests from terrestrial predators is of worldwide concern. On Queensland’s southern Sunshine Coast, depredation of turtle nests by the introduced European red fox (*Vulpes vulpes*) has been recorded as the primary terrestrial cause of egg and hatchling mortality. We investigated the impact of foxes on the nests of the loggerhead turtle (*Caretta caretta*) and occasional green turtle (*Chelonia mydas*) over ten nesting seasons. Meshing of nests with fox exclusion devices (FEDs) was undertaken in all years accompanied by lethal fox control in the first five-year period, but not in the second five-year period. Lethal fox control was undertaken in the study area from 2005 to February 2010, but foxes still breached 27% (range19–52%) of turtle nests. In the second five-year period, despite the absence of lethal fox control, the average percentage of nests breached was less than 3% (range 0–4%). Comparison of clutch depredation rates in the two five-year periods demonstrated that continuous nest meshing may be more effective than lethal fox control in mitigating the impact of foxes on turtle nests. In the absence of unlimited resources available for the eradication of exotic predators, the use of FEDs and the support and resourcing of a dedicated volunteer base can be considered an effective turtle conservation tool on some beaches.

## Introduction

The protection of sea turtle nests from terrestrial predators is a problem in many countries [1–6]. In Australia the deleterious effects of predation by the introduced European red fox, *Vulpes vulpes*, on native species are well documented [7–12]. The fox has been implicated in the decline of some native fauna, particularly those referred to as critical weight range species, weighing between 35—5500g [11, 13]. Due to this impact on Australia’s biodiversity, predation by the fox is listed as a key threatening process under the *Environment Protection and Biodiversity Conservation Act (EPBC Act) 1999*.

In 2008 the Australian government released a threat abatement plan (TAP) to provide guidelines to mitigate the impacts of fox depredation on native species. The TAP outlined five key objectives, two of which identified the need to:

- *Promote the maintenance and recovery of native species and ecological communities that are affected by fox predation*; and
- *Improve knowledge and understanding of fox impacts and interactions with other species and other ecological processes* [14].

Several biological and behavioural characteristics of the fox have supported its successful colonisation of most areas in Australia, including its ability to utilise a varied range of dietary resources and habitat types. Consequently, it is now so well established on mainland Australia that there is considered to be no chance of total eradication based on currently available methods of control [14]. Conservation efforts for native species affected by fox depredation are thus focused on mitigating the impacts of predation, rather than eradication of foxes.[13]. This is likely to continue into the foreseeable future.

**Fig 1:**
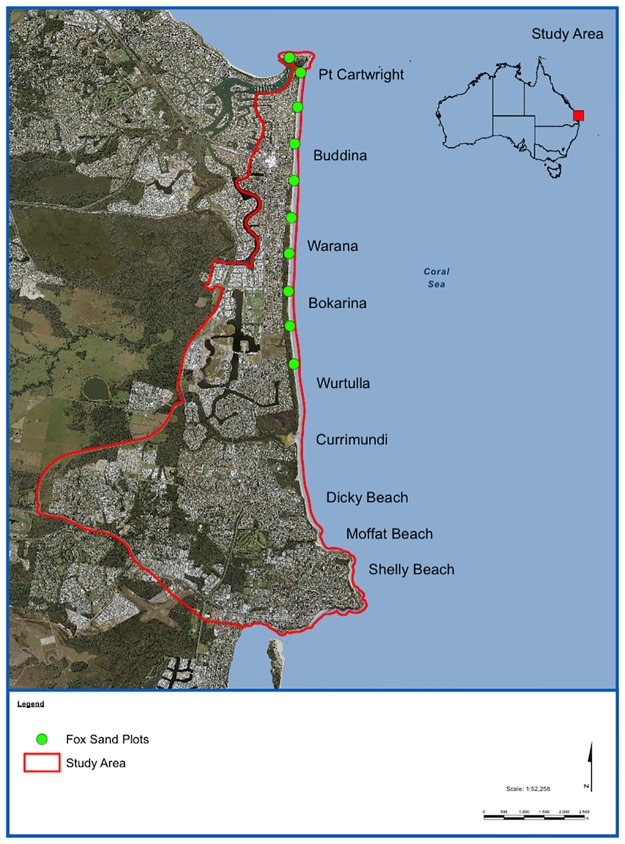
The location of the study area, in southeast Queensland, Australia (map prepared using ArcGIS 10.2, ESRI Inc).

Sea turtles lay eggs on sandy beaches around Australia’s northern coastline, including eight beaches along the heavily populated southern Sunshine Coast. The highest nest densities in this area are found at Buddina (-26.681910; 153.137811) and Shelly Beaches (-26.798886; 153.149396) at the northern and southern ends of the study area. Nesting also occurs at lower densities on Warana, Bokarina, Wurtulla, Currimundi, Dicky, and Moffat beaches (Fig 1). In eastern Australia in recent decades the fox has been the most significant terrestrial predator of sea turtles at the egg and hatchling stages [15]. Nest depredation by terrestrial predators can significantly reduce hatchling recruitment in the turtle population [16, 17]. Between 1967 and 1985 depredation of sea turtle clutches by foxes at Mon Repos, near Bundaberg (-24.804722; 152.440278), varied between ~10% to less than 0.1% [15]. Approximately 130 km further north, at Wreck Rock, depredation rates reached as high as 95% of loggerhead (*Caretta caretta*) clutches between 1976–1983 [15]. On the southern Sunshine Coast there are only anecdotal records of turtle nesting and fox activity prior to the commencement of formal monitoring in 2005, after which time continuous nesting and depredation data have been collected.

The interactions between foxes and sea turtle clutches have been well monitored and documented in the study area for the past ten years. In this study we assessed the efficacy of nest meshing and predator removal over a ten-year period, which included five years with lethal fox control and five years without lethal fox control. Our aim was to assess the influence of lethal fox control and meshing on the incidence of fox depredation of sea turtle clutches.

## Materials and methods

### Ethics statement

All turtle monitoring activities were undertaken in collaboration with and under the guidance of Queensland’s Department of Environment and Heritage Protection Threatened Species Unit staff. All activities were undertaken in strict accordance with the approval from the Department of Environment and Resource Management Animal Experimentation Ethics Committee (Queensland Turtle Conservation Project: DERM/2009/10/08-13) and the Department of Agriculture Fisheries and Forestry Animal Experimentation Ethics Committee (Queensland Turtle Conservation Project SA 2012/11/394, 395, 398, 399, 400).

### Study area

The study was conducted on Queensland’s southern Sunshine Coast on eight mostly contiguous beaches extending from Pt Cartwright (-26.679394; 153.133551), south to Shelly Beach (-26.798886; 153.149396). These beaches are all used for nesting by the loggerhead turtle and occasional green turtle (*Chelonia mydas)* (Fig 1). Wurtulla and Currimundi beaches are usually separated at all tidal stages by Currimundi Lake. Dicky and Moffat beaches were occasionally separated by Tooway Lake, but for the most part were connected by traversable beach. Sea turtle nesting in the study area is not evenly distributed across all beaches, and turtle nests occur at relatively low densities compared to some other areas of the Queensland coast [15].

The study area has a relatively short history of monitoring turtle nesting activity, with formal data collection commencing in the 2005 nesting season by a small number of community and local government volunteers. In 2007, Caloundra City Council (later to form part of Sunshine Coast Council) formally created and resourced the TurtleCare program. Under the program, local government funded a coordinator to build and support a volunteer base. The role of TurtleCare is to gather systematic data on hatching, emergence, and predation of sea turtle clutches and to protect nests from depredation by applying a protective fox exclusion device (FED) over each turtle nest. The collection of turtle nesting and clutch depredation data has increased as TurtleCare (www.turtlecare.sunshinecoast.qld.gov.au) has expanded its membership and coverage of beaches in the study area. Over the past 10 years the volunteer base has grown from around 30 in 2005 to currently 140 registered volunteers, many of whom have undertaken formal training at Mon Repos Turtle Rookery in relation to clutch relocations, post-emergence nest digs to assess incubation success, and tagging of nesting females. In addition to the registered volunteers, there is an informal network of beach walkers who report tracks and turtle sightings to TurtleCare members.

### Study design

Two impact mitigation methods were implemented: (i) lethal fox control through trapping and den fumigation; and (ii) meshing to exclude foxes from turtle nests. The study occurred over 10 nesting seasons, separated into Period 1 (2005–2009) and Period 2 (2010–2014). Lethal fox control was undertaken in Period 1, and no fox control occurred during Period 2. Meshing occurred throughout both periods.

Due to the high human population density in the study area and the associated presence of domestic dogs, both shooting and poison baiting were considered unsuitable fox control methods on beaches. Reliance, therefore, was placed primarily on den fumigation and soft-jaw trapping with subsequent destruction of trapped foxes during Period 1. The soft-jaw trapping, using a variety of food and scent lures, was generally focused on or in the vicinity of beaches experiencing disturbance of turtle nests by foxes. Fox den fumigation using carbon monoxide fumigant cartridges (DEN-CO-FUME^®^) was also undertaken in Period 1. Suspected natal dens were located through dedicated den searches and/or following fox tracks within the outlined study area. Following a final round of trapping at the end of the 2009 season, lethal fox control was suspended in the study area to allow a broader long term fox study to proceed with minimal human interference with the fox population.

**Fig 2:**
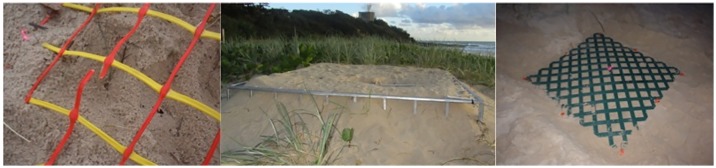
Examples of a (1) standard FED (with break), (2) aluminium FED and (3) lattice FED used to protect turtle nests from fox predation during the study.

All turtle nests discovered were fitted with a standard FED (Fig 2). The standard FED comprised a 1 m x 1 m piece of plastic mesh with 100 mm x 100 mm openings (‘Site Mesh’ OG/YW, #437824), which was laid horizontally over each nest after scraping away the top 2 cm of sand. Each FED was pegged into place using eight 30 cm-long polycarbonate pegs and then re-covered with sand. The FED was placed over each nest in a manner that ensured the centre of the FED was positioned directly over the egg chamber. Two other FED designs were used infrequently when there were concerns that the strength of the standard FED might be inadequate. The first alternative FED was a purpose built aluminium exclosure, 1 m x 1 m x 25 cm with 100 mm x 100 mm openings (Fig 2), which was used for the first time in the 2009 season. The second alternative was a lattice FED (2400 mm x 1200 mm, Premier Plastic Expanding Lattice cut to form a 1.2 m x 1.2 m FED with 90 mm x 90 mm openings) (Fig 2). Both alternative FEDs, when used, were secured in place with eight 30 cm-long polycarbonate pegs.

While the standard FED was applied to all known nests, the lattice FED was used on a small number of nests in the 2010 season for a portion of the incubation period. The lattice FED had to be removed prior to hatchling emergence due to the inability of the hatchlings to emerge en masse through openings that were smaller than those in the standard and aluminium FEDs. Since their introduction in the 2009 season, the aluminium FEDs continue to be used occasionally when foxes appear to be showing a persistent interest in a nest, and occasionally to prevent nest compaction on beaches with high human traffic.

#### Turtle nest monitoring

TurtleCare volunteers monitored nests continuously throughout the season. All disturbances, including predator impacts, hatchling emergence, and nesting success and failure data were recorded using the Queensland Turtle Conservation Project protocol [18]. We recorded the incidence of sea turtle nesting and fox depredation of sea turtle clutches for the 10 year period of the study. The nesting and emergence census in the study area was conducted each season from the date of the first nesting occurrence (usually in November) until the final natural nest emergence for the season (usually in April). Empty nests breached after emergence were noted (Table 1) but were not treated as breached. If unsure whether breach occurred during or post emergence, the nest was recorded as breached. Due to the large area of potential nesting beach relative to the size of the volunteer base, monitoring was optimised by focusing on early morning track searches and evening turtle searches, particularly when females were expected to return to lay approximately 14 days after previous nesting. Pearson *chi*-squared tests and Fisher’s Exact tests were used to assess the influence of fox control and meshing on the incidence of fox depredation of nests.

**Table 1:** Summary of meshing and number of nests breached by foxes 2005–2014 (N = 391).

	Year	Total UM	Unmeshed (UM)			Total M	Meshed (M)		
			Breached	Not breached	Breached post emergence		Breached	Not breached	Breached post emergence
Period 1	2005	8	8	0	0	18	5	13	0
	2006	9	7	2	0	26	1	25	0
	2007	7	5	2	0	22	1	19	2
	2008	7	3	2	2	40	6	33	1
	2009	13	7	5	1	56	6	46	4
Period 2	2010	1	0	1	0	33	1	30	2
	2011	0	0	0	0	25	1	24	0
	2012	5	1	3	1	51	1	49	1
	2013	1	0	1	0	28	0	28	0
	2014	5	0	5	0	36	1	35	0

#### Fox activity monitoring

Turtle nest depredation by foxes was recorded during the whole study, but other fox activity on beaches in the study area was not monitored prior to 2011. In September 2011, an annual sand plot monitoring program was implemented to track fox activity. Ten sand plots were established at approximately 1 km intervals on beaches within the study area (Fig 1) [19]. The 1.2 m wide sand plots ran perpendicularly to the beach, from the base of the first dune to the water line. Surveys were conducted annually between 2011 and 2015. During each survey, sand plots were prepared each evening between 1800 and 1900 hrs, and then checked between 0500 and 0600hrs the following morning for three consecutive days between August 30 and September 15 each year. All fox track incursions onto the sand plots were counted. A set of fox tracks heading in any direction across the sand plot was deemed an intrusion. Tracks are expressed as the number of track intrusions per sand plot per night. To enable the plots to be raked out and checked within the one-hour timeframe each evening and morning, half of the plots were used simultaneously, with the second half being operated following a break of one or two nights. A one-way ANOVA and Tukey HSD test were used to assess differences between the mean number of track incursions for each year between 2011 and 2015.

## Results

A total of 19 foxes were trapped and humanely euthanised by firearm during Period 1, and an unrecorded number of dens were fumigated. One successful den fumigation (i.e. resulting in the death of one or more den occupants) was confirmed at Moffat Beach in the 2006/7 nesting season. Despite the formal cessation of lethal fox control in June 2010, three foxes were removed from the study area in Period 2 for reasons unrelated to this study.

Although standard mesh was applied to all known nests every year in both Periods 1 and 2, in all years except 2011 a number of nests remained undetected until hatchling emergence and hence were unmeshed for the duration of incubation (range 3–31%) (Table 1). Of the 391 nests detected over the period of this study, 54 were recorded as breached by foxes during incubation and/or emergence. A further 14 nests were untouched by foxes during incubation and emergence but were disturbed after successful nest emergence (Table 1). In Period 1 when lethal fox control was being undertaken, foxes breached 27% (range19–52%) of turtle nests (Table 1). In Period 2, despite the absence of lethal fox control, the percentage of nests breached was under 3% (range 0–4%).

Depredation of sea turtle clutches was higher in Period 1 (n = 206) than in Period 2 (n = 185); X^2^ (1) = 36.39, p<0.02). In Period 1, meshing of nests provided a demonstrable level of protection against clutch depredation (X^2^ (1) = 53.86, p<0.02). In Period 2, the meshing of nests did not appear to be a factor in the low level of clutch depredation (X^2^ (1) = 1.04, p>0.05).

**Fig 3:**
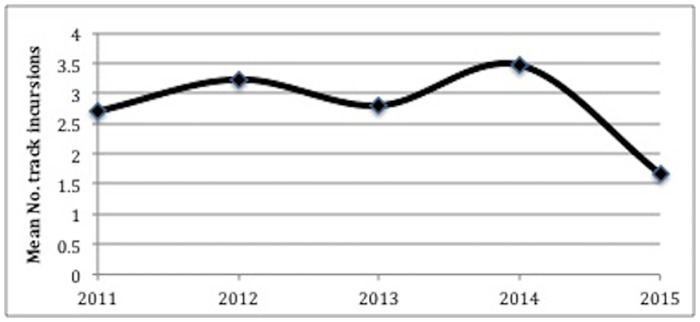
Fox activity on southern Sunshine Coast beaches, 2011–2015.

Sand plot monitoring showed that fox activity was stable across most years, but fluctuated temporally between 2012 and 2015 with a decline in fox activity in 2015 (F (4) = 4.298, p = 0.003) (Fig 3, Table 2).

**Table 2:** Post hoc comparison of the number of track intrusions on sand plots, 2011–2015, indicating where differences in fox activity can be observed between years.

(I) Date	(J) Date	Mean Difference (I-J)	Std. Error	Sig.	95% Confidence Interval	
					Lower Bound	Upper Bound
2011	2012	-.533	.473	.792	-1.84	.77
	2013	-.100	.473	1.000	-1.41	1.21
	2014	-.767	.473	.487	-2.07	.54
	2015	1.033	.473	.191	-.27	2.34
2012	2011	.533	.473	.792	-.77	1.84
	2013	.433	.473	.890	-.87	1.74
	2014	-.233	.473	.988	-1.54	1.07
	2015	1.567*	.473	.010	.26	2.87
2013	2011	.100	.473	1.000	-1.21	1.41
	2012	-.433	.473	.890	-1.74	.87
	2014	-.667	.473	.623	-1.97	.64
	2015	1.133	.473	.122	-.17	2.44
2014	2011	.767	.473	.487	-.54	2.07
	2012	.233	.473	.988	-1.07	1.54
	2013	.667	.473	.623	-.64	1.97
	2015	1.800*	.473	.002	.49	3.11
2015	2011	-1.033	.473	.191	-2.34	.27
	2012	-1.567*	.473	.010	-2.87	-.26
	2013	-1.133	.473	.122	-2.44	.17
	2014	-1.800*	.473	.002	-3.11	-.49

* The mean difference is significant at the 0.05 level.

## Discussion

This study documented reductions in fox depredation of turtle nests over a period of ten years, despite the cessation of lethal fox control after five years. The observed reduction in the depredation of turtle nests in Period 2 was somewhat counter intuitive given that one might expect a higher level of clutch depredation in the period when no lethal fox control was undertaken.

The Period 1 results showed that meshing played an important role in protecting nests from clutch depredation by foxes. Results for Period 2 appear to indicate that the reduced clutch depredation was not attributable to nest meshing. However, these results should be interpreted cautiously. The use of the FEDs appears to be beneficial in protecting turtle nests through the combination of two factors: (i) as a physical barrier preventing clutch depredation and (ii) through the indirect and longer term benefits associated with the fox’s capacity to learn and modify its foraging behavior to optimise net energy gain. All located turtle nests were covered with a standard FED as soon as possible after locating a nest. With the exception of 2012 and 2014, the known number of nests going undetected and unmeshed was declining (Table 1). Generally, since its humble beginnings in 2005, the growth of the volunteer base has resulted in fewer nests going undetected and unmeshed in most seasons.

The success of the FED program requires the capacity to minimise or eliminate opportunities for predation to enable consistent reinforcement of the “no food here” message. On occasion, this has required the use of a range of FED options to prevent clutch depredation. For example, one of the first nests of the 2011/12 season on Buddina beach was breached within one day of oviposition when a fox broke through one of the strands of the standard FED (Fig 2) and consumed a number of eggs. Given that the fox had successfully breached the FED and received some reward for its effort, it was expected that the fox would return the following night. The standard FED was immediately replaced with the sturdier lattice FED (Fig 2). As predicted, the fox returned on the night following the first depredation and attempted to breach the nest again. The stronger FED proved to be impenetrable and the nest remained intact. On the third night the fox again returned but did not attempt to dig. No further attempted nest digging occurred at Buddina beach, despite the presence of fox tracks for the remainder of the season near all the Buddina nests.

The use of the lattice FED was extended to other beaches as required in the 2011 season, and foxes did not breach any other nests that season on any beach in the study area. In this regard, the timely introduction of the impenetrable lattice FED may have contributed to foxes learning to ignore an interesting potential food odour that was consistently proving to be unobtainable. Although the lattice FEDs are no longer used, their deployment in the 2011 season may have played an integral role in the observed changes in nest predation in subsequent seasons.

A further example of the benefits of reinforcing the consistent “no food here” message may have occurred at Shelly Beach in the 2014 season. Five egg chambers were unable to be located, which resulted in all five nests remaining unmeshed for the entire incubation period. No predation occurred on any of the nests despite the beach falling within the home range of a known and monitored fox family. In this instance, the five unmeshed nests functioned as unintended control nests. While the most conclusive test would involve the removal of mesh from all nests, this would be inappropriate for two reasons; i/ the loggerhead turtle is listed as Endangered under Queensland’s *Nature Conservation Act 1992* and Australia’s federal *EPBC Act 1999*; and ii/ deliberate mesh removal could be perceived as undermining the considerable conservation efforts of the TurtleCare volunteers.

Despite the strength of the lattice FED, it is impractical for routine use due to the difficulty transporting it (compared to the standard FED, which can be rolled) and the requirement for its removal prior to nest emergence due to hatchling inability to pass through easily en masse. The aluminium FED (Fig 2) was also an available option where foxes appeared to be showing increased interest in a nest meshed with a standard FED. The aluminum FED also has the problem of being difficult to transport, but has the advantage that hatchlings can easily pass through during emergence, which enables it to be left in place up to and beyond hatchling emergence.

Without fox density data, and with the fox activity index only commencing in 2011, one cannot discount the possibility that the lethal fox control in Period 1 contributed to a reduction of the fox population in Period 2. However, we believe this is unlikely for two reasons: i/ fox tracks continued to be found during Period 2 near most nests at all beaches; and ii/ other components of the fox study commenced in 2010 confirmed the presence and relative stability of fox populations across the five years of Period 2 (J. O’Connor, unpublished).

Danchin et al, p701 define learning as “an adaptive change in behaviour through the effect of acquired information, which provides experience” [20]. Virtually all animals have the capacity to learn from experience, which enables them to modify their behaviour in response to situations they could not experience before birth [21]. Avital and Jablonka [22] note that the ability to learn allows animals to create new and discard old behaviour patterns in response to changing, but recurring, features in their environment. Ultimately, learned behaviour enables an animal to use its physiological traits to maximise its fitness. In some instances it may be in response to an acute life or death situation, such as avoiding a predator, but learned experience is also likely to teach it where to best invest its energy for greatest reward. Also, like many other species, foxes are thought to be adept at teaching their young a range of life skills, including where and how to obtain food [5, 23]. Thus, learned behavior in relation to which cues are likely to provide an energy benefit, and which are not, could be passed from parent to offspring. This may explain the cross-generational decline in nest depredation found in this study despite the consistent presence of a fox population.

Such learned behavior and rationalisation of investment has been well documented in other species too. Sherry [24] noted the black-capped chickadee’s (*Poecile atricapillus)* capacity to recall which of its caches had been retrieved, which enabled it to avoid wasting time and energy on a site that would yield no reward. A recent study on the use of cages to protect white-fronted chats (*Epthianura albifrons*) found that 85% of attempted predation events, mostly from corvids, lasted less than two minutes when the potential predator was unable to breach the protective cage [25]. In his comprehensive study in Canada’s boreal forests, David Henry also documented the intricate system of urine marking that red foxes used to optimise energy expenditure during foraging [26].

Studies in Turkey and the USA have also documented the successful use of predator exclusion mesh and cages to reduce depredation of sea turtle nests by foxes [6, 27]. Ratnaswamy et al [28] found that the meshing and caging of sea turtle nests more effectively reduced depredation by raccoons than lethal predator control. Some studies, however, have demonstrated that exclosures may actually lead to increased predation [29–34], probably in most cases prompted by the visual stimulus of the potential prey inside the exclosure. For nest exclosures in the study area, the location of the FED below the surface of the sand eliminates, or greatly reduces, the opportunity for discovery through visual cues. The irregularity of nest emergence also makes it impractical for a predator to wait for a turtle nest emergence to access hatchlings. For example, incubation in the study area takes on average 69 days but with a range of 51–93 days (n = 155). The fox is thought to use mainly olfactory cues, such as turtle body scent on turtle nesting crawls and egg odour to locate turtle nests [35] and some visual clues [34]. This may explain why foxes only occasionally dug under the outside edges of the mesh but more often persisted in the centre above the chamber where the odour was likely to be strongest.

The success of nest exclosures elsewhere for other species has been variable. In Alaska, where predator exclosures were used to protect nesting western sandpipers (*Calidris mauri*), successful exclusion of arctic foxes (*Vulpes lagopus*) decreased as the nesting season progressed [32]. The persistence of the arctic fox in that study may have been fueled by the additional visual stimulus of sandpipers inside the exclosure. An incubating turtle nest provides olfactory stimuli, and probably auditory stimuli in the few days prior to emergence, but no visual stimuli until the hatchlings emerge.

The use of nest exclosures, or FEDs, as a primary conservation tool is not suitable for all locations. In many instances the use of predator exclosures may be impractical due to a range of factors, including cost, nesting density, or remoteness. For example, meshing of turtle nests in the manner described would be impossible to implement at Mon Repos, eastern Australia’s largest mainland loggerhead turtle rookery. Meshing for predator control on that beach would require the use of more than 1,000 pieces of mesh spread across only 1.6km of beach, with 50% of them concentrated in less than 300m. In such conditions, the mesh and pegs would become obstacles for nesting turtles and potential hazards for other beach users.

There is a range of tools available to control foxes and mitigate their impacts on vulnerable native species. This study has shown that non-lethal fox management can increase hatching success in sea turtle clutches. We also suggest that over time, meshing changes fox foraging behaviour on turtle nesting beaches (i.e reduced incidence of nest digging). We suggest that the use of FED’s to mitigate fox impacts works well in the study area for a number of reasons: turtle nesting density is relatively low, the fox population appears to be quite stable, and financial and human resources have been committed to turtle conservation on the Sunshine Coast. In relation to the latter, the Sunshine Coast nesting beaches occur fortuitously directly adjacent a large urban population, which is home to a dedicated volunteer base. The fostering of conservation volunteerism provides a valuable means of combining community engagement, ecological research and education to achieve conservation outcomes that might otherwise be unachievable.
